# *TGFBR1* Variants Can Associate with Non-Syndromic Congenital Heart Disease without Aortopathy

**DOI:** 10.3390/jcdd10110455

**Published:** 2023-11-09

**Authors:** Manal Alaamery, Nour Albesher, Fahad Alhabshan, Phil Barnett, Mohamed Salim Kabbani, Farah Chaikhouni, Aho Ilgun, Olaf R. F. Mook, Hessa Alsaif, Vincent M. Christoffels, Peter van Tintelen, Arthur A. M. Wilde, Arjan C. Houweling, Salam Massadeh, Alex V. Postma

**Affiliations:** 1 Developmental Medicine Department, King Abdullah International Medical Research Center, King Saud bin Abdulaziz University for Health Sciences, King Abdulaziz Medical City, Ministry of National Guard—Health Affairs, Riyadh 11481, Saudi Arabia; 2 Saudi Genome Program, National Centre for Genomic Technologies, King Abdulaziz City for Science and Technology (KACST), Riyadh 11442, Saudi Arabia; 3 KACST-BWH Centre of Excellence for Biomedicine, Joint Centres of Excellence Program, King Abdulaziz City for Science and Technology (KACST), Riyadh 11442, Saudi Arabia; 4 Department of Biological Sciences, Faculty of Sciences, King Abdulaziz University, Jeddah 21589, Saudi Arabia; 5 Department of Cardiac Sciences, Ministry of the National Guard—Health Affairs, King Abdullah International Medical Research Center, King Saud bin Abdulaziz University for Health Sciences, Riyadh 11481, Saudi Arabia; 6 Department of Medical Biology, Amsterdam University Medical Centre, 1105 AZ Amsterdam, The Netherlands; p.barnett@amsterdamumc.nl (P.B.); a.ilgun@amsterdamumc.nl (A.I.);; 7 Department of Human Genetics, Amsterdam University Medical Centre, 1105 AZ Amsterdam, The Netherlands; o.r.mook@amsterdamumc.nl (O.R.F.M.); a.houweling@amsterdamumc.nl (A.C.H.); 8 Department of Genetics, University Medical Center Utrecht, Utrecht University, 3584 CS Utrecht, The Netherlands; j.p.vantintelen-3@umcutrecht.nl; 9 Department of Cardiology, Amsterdam University Medical Centre, 1105 AZ Amsterdam, The Netherlands; a.a.wilde@amsterdamumc.nl

**Keywords:** congenital heart defect, TGFBR1, whole-exome sequencing, gain of function, TGFbeta-smad signaling

## Abstract

Background: Congenital heart diseases (CHD) are the most common congenital malformations in newborns and remain the leading cause of mortality among infants under one year old. Molecular diagnosis is crucial to evaluate the recurrence risk and to address future prenatal diagnosis. Here, we describe two families with various forms of inherited non-syndromic CHD and the genetic work-up and resultant findings. Methods: Next-generation sequencing (NGS) was employed in both families to uncover the genetic cause. In addition, we performed functional analysis to investigate the consequences of the identified variants in vitro. Results: NGS identified possible causative variants in both families in the protein kinase domain of the TGFBR1 gene. These variants occurred on the same amino acid, but resulted in differently substituted amino acids (p.R398C/p.R398H). Both variants co-segregate with the disease, are extremely rare or unique, and occur in an evolutionary highly conserved domain of the protein. Furthermore, both variants demonstrated a significantly altered TGFBR1-smad signaling activity. Clinical investigation revealed that none of the carriers had (signs of) aortopathy. Conclusion: In conclusion, we describe two families, with various forms of inherited non-syndromic CHD without aortopathies, associated with unique/rare variants in *TGFBR1* that display altered TGF-beta signaling. These findings highlight involvement of *TGFBR1* in CHD, and warrant consideration of potential causative *TGFBR1* variants also in CHD patients without aortopathies.

## 1. Introduction

Congenital heart disease (CHD) is the most common congenital malformations in newborns, and it remains the leading cause of mortality among infants under one year old [1]. CHD consists of a cluster of structural abnormalities, ranging from insignificant defects to complex life-threatening malformations. Although significant progress has been made in understanding CHD, the actual etiology of many CHDs is unknown yet [2].

Studies have shown that CHD is a multifactorial disorder induced by both genetic predisposition and environmental influences. CHD is also a genetic heterogeneous disorder as it can be caused by both chromosomal abnormalities, such as chromosomal deletions and duplications, as well as single-gene defects. In most cases, CHD occurs in an isolated, non-syndromic form; however, it sometimes occurs in syndromic forms often with extracardiac anomalies [3]. Most congenital heart defects are sporadic, although an increasing number of familial cases with various types of CHD have been reported recently [2]. A recent study has shown that co-occurrence of CHD malformations in families is caused by shared susceptibility genes [4]. Advancements in genomic sequencing technologies have demonstrated great potential in delineating many risk loci associated with CHD susceptibility and proved helpful in improving our molecular understanding of cardiogenesis and normal heart development.

Here, we report on two families suffering from a spectrum of CHD phenotypes: one family is a large Dutch family with an autosomal dominant inheritance, and the other a consanguineous family is from Saudi Arabia with an autosomal-recessive inheritance. In the first family, the affected have various forms of congenital heart disease coupled with an abnormal atrial rhythm and conduction disturbances. Affected individuals from the second family showed depressed cardiac function and common atrioventricular valve regurgitation. Targeted and whole-exome sequencing (WES) was performed, and a unique variant in the *TGFBR1* was identified in both families. Interestingly, in both families, the rare variant hit the same amino acid; however, this resulted in two different substituted amino acids. Both variants co-segregated with the disease according to the expected inheritance patterns. Moreover, both variants demonstrated altered TGFBR1-smad signaling activity. Taken together, we illustrate that variants in *TGFBR1* can also lead to familial forms of non-syndromic congenital heart disease without aortopathy.

## 2. Materials and Methods

### 2.1. Ethics Statement

Informed consent was obtained from all subjects and/or legal guardians. For minors, written informed consent was obtained from their parents. The study design and protocol were approved by the Institutional Review Board (IRB) at King Abdullah International Medical Research Center (KAIMRC), Ministry of National Guard-Health Affairs (MNGHA), and followed Helsinki protocols.

### 2.2. Extraction of Genomic DNA

Genomic DNA was extracted using the QIAquick DNA extraction kit (QIAamp, Qiagen, Valencia, CA, USA). DNA quantity and quality were assessed using the Nanodrop-2000^TM^ spectrophotometer (Thermo Scientific, Schaumburg, IL, USA).

### 2.3. Targeted Resequencing Family A

An amount of 5 μg of DNA was fragmented according to the manufacturer’s instructions (Covaris, Woburn, MA, USA). DNA quality was assessed using a Bioanalyzer (Agilent, Santa Clara, CA, USA). The sample was barcoded using standard Roche protocols. Libraries were amplified by linker-mediated PCR. Samples were mixed equimolarly (3 μg in total) and loaded onto the capture array according to the manufacturer’s instructions (Nimblegen, Basel, Switzerland). To increase target enrichment, samples were hybridized a second time on the same array. The enriched library was diluted, annealed to capture beads, and clonally amplified by emulsion PCR. After emulsion PCR, beads with clonal amplicons were enriched and deposited on a picotiter plate and sequenced on the GS FLX Titanium (Roche Diagnostics GmbH, Roche Applied Science, Mannheim, Germany). The array was custom designed to include the coding exons of the 402 genes of the linked chromosome 9q region, including 100 bp flanking intronic sequences. All variants of interest were confirmed by Sanger sequencing.

### 2.4. Whole-Exome Sequencing (WES) Family A

WES and subsequent bioinformatics filtering steps were carried out as described earlier on individual IV-10 using an Illumina platform.

### 2.5. Chromosomal Microarray Analysis (CMA) Family B

CMA was commercially performed (Centogene, Rostock, Germany). Proband’s genomic DNA was fragmented, amplified, and hybridized to the Cytoscan^TM^ HD array (Affymetrix) according to the manufacturer’s protocol. The results were analyzed with the Chromosome Analysis Suite (ChAS, Affymetrix, Santa Clara, CA, USA). Copy number variations (CNV) with a minimum of 25 markers and a size of more than 50 kb (deletions) and 200 kb (duplications) are reported.

### 2.6. Whole-Exome Sequencing (WES) Family B

WES was carried out by Centogene (Centogene: Rostock, Germany) for all family members consisting of two affected siblings (proband: II:1, II:2) and both parents (I:1, I:2). Full exome capture and sequencing were performed using an Illumina platform. The sequencing libraries were enriched for target regions using the Twist Human Core Exome Plus kit. The captured libraries were sequenced to obtain at least 20x coverage depth for >98% of the targeted bases.

### 2.7. Annotation and Filtering of Disease-Causing Variant

Data analysis and interpretation were performed by Centogene using an end-to-end in-house bioinformatic pipeline, including read alignment to GRCh37/hg19 ((GRCh37; http://genome.ucsc.edu/ accessed on 29 of November 2019) genome assembly, variant calling, annotation, and comprehensive variant filtering. The analysis pipeline has been previously described. Following primary filtration of low-quality reads and possible artifacts and variant annotation, detected variants were screened using several databases, including gnomAD, HGMD, ClinVar, and CentoMD. All variants with minor allele frequency (MAF) of less than 1% are considered. The investigation for a relevant disease-causing variant is focused on coding exons and flanking +/−20 intronic nucleotides of genes with clear gene–phenotype evidence (OMIM information), considering all potential modes of inheritance patterns. In addition, family history and clinical information are used to evaluate identified variants with respect to their pathogenicity and causality. Only variations within genes potentially related to the proband’s medical condition were reported.

### 2.8. Plasmids and Generation of TGFBR1 Mutants

The full-length wild type, CA human TGFBR1/ALK5 cDNA (pcDNA3.1+, NM_004612.2), and CAGA 9-MLP-Luciferase Reporter Vector [5] (pGL3Basic) were obtained from Maarten van Dinther (LUMC, Peter ten Dijke Lab.). The R398C, D400G, and R398H variants were introduced into the wild-type construct by PCR-based site-directed mutagenesis and verified by sequencing and digestion.

### 2.9. Cell Culture and Transfection

HEK 293T cells (or 3T3 cells) were cultured in DMEM 15-013CV (Corning, Somerville, MA, USA) supplemented with 10% heat-inactivated FBS (Sigma F7524, Lot BCCB1938, PT Merck Chemicals and Life Sciences. Merck Life Science NV, Amsterdam, The Netherlands), 2 mM glutamine, 100 units/mL penicillin, and 100 µg/mL streptomycin in a 12-well (or 24-well) culture plate, in a humidified incubator at 37 °C with 5% CO_2_. Transfection with wild-type and mutant TGFBR1 plasmids was performed as follows. The day before transfection, cells were seeded at a density of 80–90 × 10^3^ (or 65–70 × 10^3^) cells per well containing 1 mL (or 0.5 mL) culture medium. The next day, transient transfections were performed at the end of the day in triplicate using polyethylenimine (25 kDa, linear, Brunschwig Chemie BV, Amsterdam, The Netherlands). Ratio DNA (µg): PEI (µg) = 1:3 (or 1:4). In short, per well, 450 ng (or 150 ng) TGFBR1 plasmid was co-transfected with 450 ng (or 750 ng) CAGA 9-MLP-LUC and 100 ng wild-type Renilla (pGL3Basic). Separately, a PEI and a DNA cocktail in 50 µL sodium chloride solution (150 mM) were prepared. Then, the PEI cocktail was drop-wise added to the DNA cocktail under mild vortexing (25–30 s) and left for 30–45 min at RT (20–25 °C) to form the necessary cationic (PEI)/DNA complexes. Subsequently, this cocktail was added drop-wise to the cells, followed by a homogenization step by gently rocking the plate. After overnight incubation, the medium was removed early the next morning, and cells were washed once with 1X PBS. Subsequently, fresh culture medium was added (1 mL/well). At 40–48 h (or 20–24 h) post-transfection, cells were lysed for luciferase assay.

### 2.10. Luciferase Assay

The CAGA 9-MLP-Luciferase reporter was used, and Renilla served as normalization control. Luciferase activity was assayed with a GloMax Explorer Multimode Microplate Reader (Promega, Madison, WI, USA). All conditions were transfected and measured in three independent wells per transfection session, and this was repeated three times, for a total of n = 9 independent measurements per condition. Renilla-normalized values per condition (caga-luc, WT, R398C, R398H, D400G, and T204D) were factor corrected for intersession variation as described [6]. An ANOVA analysis (Kruskal–Wallis) with a S-N-K post-hoc test was run on the values using Sigmastat (Sigmastat 3.5, Systat Software, San Jose, CA, USA). Differences per condition were deemed significant when the *p*-value was below 0.05. Cell Lysates were snap frozen and incubated at −80 °C until ready for Western blot analysis.

### 2.11. Western Blot

TBST: 50 mM Tris, 150 mM NaCl, 0.1% Tween-20. Milk Powder Solution (MPSOL, Sydney, Australia): 5 and 1% Protifar (Nutricia, Gaithersburg, MD, USA) in TBST. Membrane: PVDF membrane, Immobilon-P, Millipore (Burlington, MA, USA). Antibodies: rabbit anti-TGFBR1 (sc-398, V-22) and donkey anti-rabbit-HRP (GE Healthcare, NA930V), goat anti-rabbit-AP (Dako, D0487) 1:5000. hTGFBR1 expression was checked by Western blot analysis of 20 µL luciferase lysate according to standard procedures. Briefly, proteins were separated by SDS-PAGE (10% gel). Transferred to the membrane using the semidry blotting technique. Subsequently, the membrane was blocked at 4 °C for 2–3 h in MPSOL (5%). Thereafter incubated with the anti-TGFBR1 antibody in MPSOL (1%, 1:200) at 4 °C for 20–24 h. The next day, the membrane was washed 3 times quickly and 3 times for ~20 min with TBST before the membrane was incubated with the secondary antibody donkey anti-rabbit-HRP or goat anti-rabbit-AP in MPSOL (1%, 1:5000) at RT for ~2 h. Subsequently, the membrane was washed 3 times quickly and 3 times every ~20 min with TBST prior to detection. Detection: ECL Select (GE Healthcare) for 5 min according to the manufacturer’s procedure or NBT/BCIP (Roche, 1:50 in AP-buffer) according to the manufacturer’s procedure. The membrane was imaged using the ImageQuant™ LAS 4000 imager (GE Healthcare, Chicago, IL, USA). For the Western blot, three luciferase samples were pooled per condition. We only performed Western blot (as an independent means to check transfection efficiency) for the last transfection experiment, as all Renilla-corrected measurements per condition across the three transfection experiments were similar.

### 2.12. qPCR

Total RNA was isolated using the Nucleospin RNA II kit (Bioke/Macherey Nagel, Düren, Germany) following the manufacturer’s recommendations with the following adaptations. A 600 µL lysis solution containing 10 mM TCEP was added to each well. Lysis was performed by shaking on a standard plate shaker at RT and 1200 rpm for approximately 30 min. DNAse treatment was performed on columns for 30–45 min. At each step, column load (sample, buffers, etc.) was left for 3–5 min prior to centrifugation. RNA was eluted from the column with 70 µL water. RNA concentration was measured on the NanoDrop 1000. As an indication, measured concentrations varied from ~500 to 900 µg/µL (A260/A280 ≥ 2.10 and A260/A230 ≥ 2.2). First-strand cDNA was synthesized from this isolated total RNA (1 µg for both the + RT and -RT reaction) using oligo(dT) primer and the Superscript II RT-PCR kit (Invitrogen, Waltham, MA, USA) in a 25 µL reaction according to the manufacturer’s procedure. Quantitative PCR was performed on a LightCycler480 (Roche). Amplicon inputs (ng fs cDNA): 15. Annealing temperature was 60 °C (for all amplicons). Data were analyzed with LinRegPCR. The average/mean PCR efficiency was subsequently used for calculating the average mRNA copy number. mITGB1 (mouse Integrin beta-1) expression levels were used to correct for variations in RNA input. Values are expressed as average ± SD, * statistically significant difference versus wild type; # versus vector (*p* < 0.05).

## 3. Results

We report here on two families with various forms of congenital heart disease, their clinical and genetic work-up, and the resultant findings.

**Figure 1:**
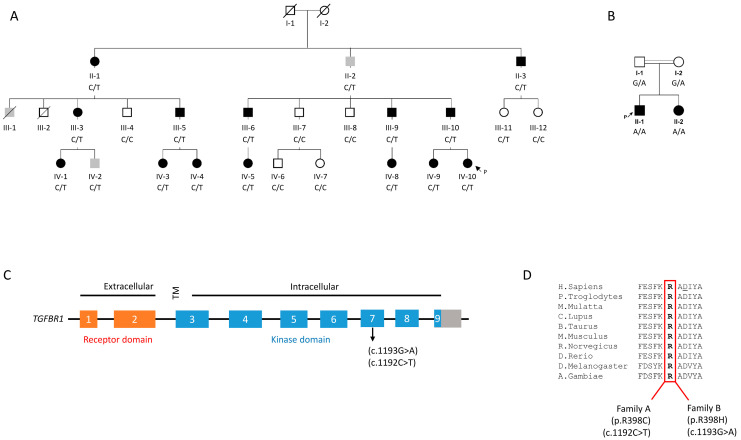
(**A**) Pedigree of Family A with an autosomal dominant mode of inheritance. Females and males are indicated by circle and square symbols, respectively. Clear symbols denote healthy individuals, and filled black symbols denote affected individuals; gray symbols indicate affected retrospectively based on clinical records or individual only had negative p wave on ECG. Heterozygous carriers of the (c.1192C > T; p.R398C) TGFBR1 variant are represented by C/T; arrow indicates the proband. (**B**) Pedigree of Family B depicting an autosomal-recessive mode of inheritance. Homozygous and heterozygous states of the (c.1193G > A; p.R398H) TGFBR1 variant are represented by A/A and G/A symbols, respectively. Arrow indicates the proband. (**C**) Schematic depiction of the TGFBR1 protein, its exons, and the location of the two variants herein. TM denotes transmembrane. (**D**) Multiple protein sequence alignment of Arg398 across different species showing the high degree of conservation of arginine at position 398 and its immediate surroundings. Both R398 TGFBR1 variants demonstrate altered SMAD signaling.

Family A is a large four-generation Dutch family with an autosomal dominant inheritance of which the clinical details and linkage analysis have been described earlier in a publication from 2011 [7]. A simplified version of the pedigree of Family A is depicted in Figure 1A. In short, the clinical phenotype of the affected is characterized by various forms of congenital heart disease, including atrial septal defects, tetralogy of Fallot, and persistent left superior vena cava. In addition, many of them also have an abnormal atrial rhythm and conduction disturbances, while those in the oldest generation only have the latter. For further phenotypic details, we refer back to the original publication [7] and Supplemental Table S1. We have performed additional clinical investigations to determine whether any abnormalities of the aorta were present in carriers of the linked chromosome 9q haplotype (see below). For Family A, we were able to retrieve the clinical records of 28 family members. A total of 17/28 were carriers of the p.R398C variant, and for 8/17 variant carriers, we were able to retrieve an ultrasound or MRI report in which measurements of the aorta were recorded and the age at evaluation (Supplementary Table S2). These clinical records were evaluated by experienced, established clinicians (ACH, PvT). None of the carriers had a dilation of the aorta or mention of a previous aortic aneurysm or dissection, as shown in Supplemental Table S2. Moreover, for the carriers in which no ultrasound of the aorta was described, there were also no references to an aneurysm in available clinical records.

Regarding the genotype, in the original publication, chromosomal abnormalities were ruled out by means of an array CGH. In the 2011 publication, linkage analysis was performed, and only one locus on chromosome 9q reached genome-wide significance with a LOD score of 3.3 [7]. The identified locus was 39 Mb large and contained 402 genes. Several candidate genes were Sanger sequenced then, including TGFBR1, as one of the presumed candidate genes for the phenotype, though no pathogenic variants were identified in any of the candidate genes at that time, as published [7]. However, as this region is quite gene dense and screening technologies have improved tremendously with the introduction of next-generation sequencing, we subsequently switched to a targeted next-generation resequencing approach. In this way, we screened the coding sequence of all 402 genes in the linkage area in two affected patients (IV-3, IV10). By using standard bioinformatic filtering criteria based on the frequency and consequences of the variant, we identified a single, protein-altering variant of uncertain significance, present in both investigated affected individuals. There were no other rare, shared splice sites, or protein-altering variants identified. The variant occurs in *TGFBR1* (c.1192C > T) and leads to a missense change, resulting in the substitution of Cys for Arg at amino acid position 398 (p.R398C) within the protein kinase domain of TGFBR1 (Figure 1C, Supplemental Table S4). Subsequent familial segregation analysis of this variant by Sanger sequencing demonstrated that it co-segregated with the phenotype within the family in the expected autosomal dominant fashion, except for Individual III-11, who was a carrier of the variant while being unaffected (Figure 1A). To corroborate this, we also performed additional whole-exome sequencing in IV-10, but we did not identify additional pathogenic variants in genes that are established in causing cardiac, arrhythmic, or aortic disease, except for the TGFBR1 variant. The variant is absent from the Genome Aggregation Database (gnomAD) with over 250 k (presumed) healthy controls [8]. The amino acid Arg398 and its surroundings are strongly evolutionarily conserved (Figure 1D). Moreover, TGFBR1 missense variants are exceedingly rare in gnomAD in general, especially around Arg398, indicating that variation (here) is not well tolerated (Supplemental Table S4).

**Figure 2:**
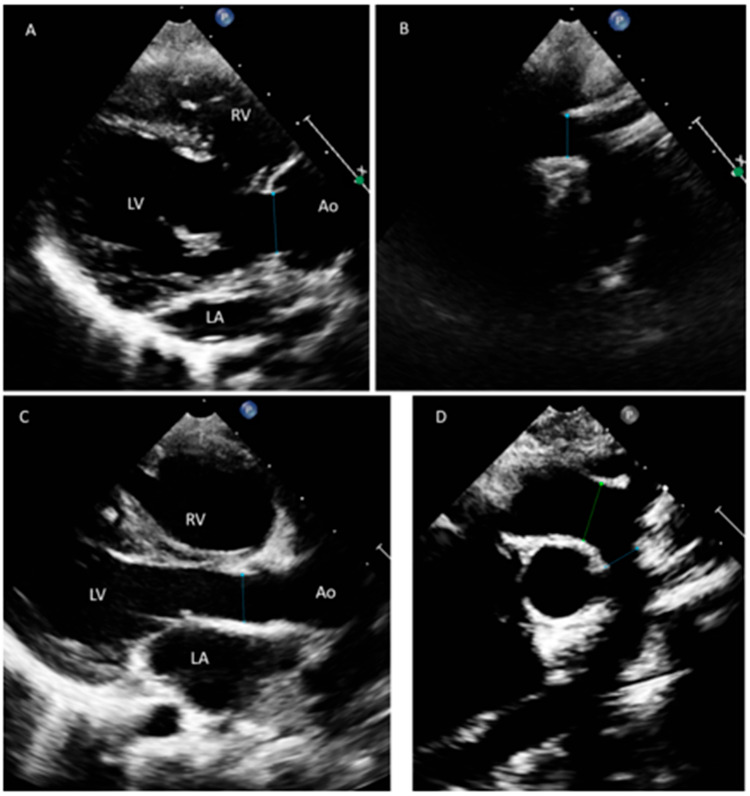
Echocardiographic views taken for both Saudi siblings at the parasternal long-axis views (**A**,**C**) and suprasternal sagittal views (**B**,**D**). Both siblings have normal measurement and z score for the aortic valve and aortic arch. The first sibling has normal aortic valve (**A**) that measures 1.4cm (z score 0.3) and normal transverse aortic arch 1.2cm (z score 0.3) (**B**). The other sibling has normal aortic valve (**C**) that measures 1.0cm (z score -1) and normal transverse aortic arch 0.8cm (z score—1.4) (**D**). Ao; aorta, LA; left atrium, LV; left ventricle, RV; right ventricle.

In Family B, the proband patient is a 7-year-old male (II-1, Figure 1B) and the first of a Saudi consanguineous family. The proband was diagnosed with various forms of CHD specifically: a large atrial septal defect (ASDII), a large inlet ventricular septal defect (VSD), a dysplastic and straddling mitral valve, tricuspid valve hypoplasia, and right ventricular hypoplasia. He had undergone several surgical procedures, including pulmonary artery (PA) banding and reconstruction of the main PA with homograft. The proband’s sister (II-2), 21 months old, also had a confirmed diagnosis of CHD. She suffered from depressed cardiac function and moderate common atrioventricular valve regurgitation and was identified with complete atrioventricular septal defect (AVSD) with common atrium, and she had undergone a PA banding surgery (for details, see Supplemental Table S3). Both siblings belong to healthy parents (I-1, I-2) who have no family history of cardiac anomalies. Aortic abnormalities were also investigated in this family, but there was no evidence of abnormal aortic widening or dissection, nor were there any reports of sudden deaths. Both siblings had a normal measurement and z score for the aortic valve and ascending aorta (Figure 2).

Given the inheritance and the shared phenotype in the two children, we sought to determine if a genetic defect was associated with the clinical presentation of the index patient. Initially, chromosomal microarray analysis (CMA) was performed in Family B to rule out genomic imbalances; CMA analysis did not detect aberrant CNVs. Subsequently, we performed whole-exome sequencing (WES) for all Family B members consisting of the two affected siblings (II-1, II-2) and both parents (I-1, I-2). The WES analysis in Family B revealed a variant of uncertain significance in the *TGFBR1* gene. This *TGFBR1* variant, c.1193G > A, which causes results in a missense variant, changing an Arg to His at position 398 (p.R398H), within the protein kinase domain of TGFBR1 (Figure 1C), which is the only protein-altering variant that is patient-shared and segregating with the family pedigree. Subsequent familial segregation analysis using Sanger sequencing demonstrated that this variant co-segregates with the familial phenotype with an autosomal-recessive mode of inheritance (Figure 1B). Both affected siblings are homozygous for the identified variant, while both parents are heterozygous carriers (Figure 1A). This variant is present only once in over 250k control alleles in a heterozygous state in gnomAD [8]. As mentioned for Family A, the amino acid Arg398 and its surroundings are strongly evolutionarily conserved (Figure 1D), and TGFBR1 missense variants in the general population at this location are exceedingly rare (Supplemental Table S4).

To identify other patients with rare TGFBR1 variants, we checked available local CHD cohorts for which we had exome or genome data (WES/WGS). In total, we had data available of 412 patients (75 Ebstein anomaly, 127 tetralogy of Fallot, 210 transposition of the great arteries). No other rare TGFBR1 variants (MAF < 1 in 1000 autosomal recessive or <1 in 10,000 autosomal dominant) were identified in these individuals.

**Figure 3:**
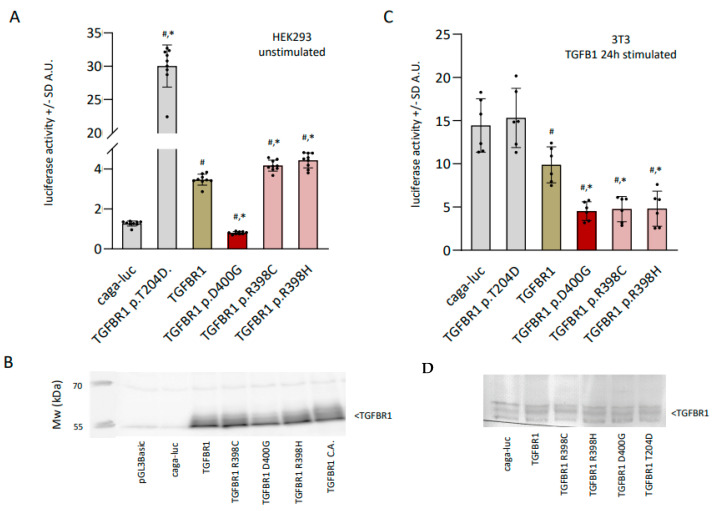
(**A**) Relative activation of luciferase in various conditions in HEK293 cells using caga-luc as a readout (caga-luc, TGFBR1 T204D, wild-type TGFBR1, TGFBR1 p.D400G, TGFBR1 p.R398C, TGFBR1 p.R398H). A.U. arbitrary units, T204D is a constitutively active form of TGFBR1. * *p* ≤ 0.05 in relation to WT TGFBR1, # *p* ≤ 0.05 in relation to caga-luc only, n = 9 per condition. (**B**) Representative Western blot of one transfection experiment demonstrating the transfection efficiency; three pooled samples were used per condition. (**C**) Similar experimental set-up as in Figure 2A, except in 3T3 cells that were stimulated for 24 h with TGFB1. n = 6 per condition. (**D**) Representative Western blot of one transfection experiment demonstrating the transfection efficiency; three pooled samples were used per condition.

Given that both variants occurred in the protein kinase domain of TGFBR1, we wanted to assess the consequences of the R398 variants on the function of TGFBR1. Specifically, we wanted to investigate the impact of the variants on SMAD signaling, which is one of the primary downstream effector pathways of TGFBR1. To this end, we employed an established luciferase assay using a Smad binding elements (SBE)-luc plasmid (CAGA-luc in Figure 3), known to capture SMAD signaling downstream of TGFBR1 [9]. These in vitro experiments were performed in unstimulated HEK293T cells [10] and in TGFB1-stimulated 3T3 cells, a mouse fibroblast cell line commonly used in TGF-β/Smad signaling. We measured several conditions, including a known Loeys–Dietz variant (p.D400G), a known constitutively active TGFBR1 variant (p.T204D), and the variants of the two families (p.R398C, p.R398H, n = 9 or n = 6 biological replicates). In HEK293 cells, statistical analysis (ANOVA) demonstrated that there were significant differences between the various conditions. A post-hoc analysis demonstrated differences between all the conditions, except between p.R398C and p.R398H (Figure 3A). In short, the caga-luc vector demonstrates that there is SMAD activity detected in HEK293T cells (background SMAD activity of the cells), indicating that TGFBR1 signaling is active and present (Figure 3A). This is corroborated by our Western blot, using a TGFBR1 antibody, showing a faint band in caga-luc-transfected cells at the right molecular weight of TGFBR1 (Figure 3B). As a positive control, we co-transfected a constitutively active form of TGFBR1 (p.T204D) [11], which demonstrated a strong and significant increase of luciferase activity (30×) above the caga-luc vector only, indicating the maximum range of Smad signaling that could be detected in this assay in this cell line. As a negative control, we tested the established pathogenic Loeys–Dietz syndrome (LDS) p.D400G TGFBR1 variant [12], which occurs only two amino acids away from our variants, and is characterized as loss of function. Indeed, in our assay, we observed a significant loss of activity for the p.D400G variant of approximately 50% in comparison to wild-type TGFBR1 (Figure 2A). Then, we compared wild-type TGFBR1 with both the p.R398C TGFBR1 and p.R398H TGFBR1 plasmids. Both the p.R398C and p.R398H variants showed a modest but significantly increased activity above wild-type TGFBR1 (Figure 3A). There was no difference in activity between the two R398 variants. We conclude that both the p.R398C and p.R398H TGFBR1 variants are gain of function, in contrast to earlier published (LDS) TGFBR1 variants in this cell line. As HEK293 cells are less ideal for testing SMAD signaling, we also performed similar experiments in a TGFB1-stimulated 3T3 cell line, commonly used in TGFB pathway research. Indeed, in the 3T3 cell line, we saw a different response. The basal background SMAD activity (caga-luc) was present at higher levels, as expected, but could not be increased by the constitutively active variant (T204D), likely indicating that the cells were maximally stimulated in this condition (Figure 3C). Transfecting wild-type TGFBR1 in this system significantly lowered SMAD activity compared to baseline (caga-luc), likely reflecting a feedback mechanism or modulation of the pathway. Interestingly, upon transfecting either the R398 variants or the p.D400G LDS variant, the SMAD activity was significantly lowered in comparison to wild-type TGFBR1, in effect displaying a loss of function (Figure 3C). Transfection efficiencies were equal for all conditions (Figure 3D and Supplemental Figure S1A). We conclude that in stimulated 3T3 cells, both our p.R398C and p.R398H TGFBR1 variants behave similarly to the known pathogenic LDS TGFBR1 variant, and all of them significantly differ in their response compared to wild-type TGFBR1. We also checked whether these differences could be observed for phosphorylated smad2/3 using a Western blot (Supplemental Figure S2). However, we do not observe a difference in p-smad2/3 across the conditions, likely the results of the already high p-smad2/3 present at baseline and the fact that Western blots are less sensitive to pick up such differences. There might be a trend of increased p-smad2/3 in the 24 h stimulated condition; however, no differences between the R398 variants and wild type can be observed. Lastly, we investigated several genes involved in TGFB signaling, which are expressed in 3T3 cells, using qPCR. Here, we observe a significantly increased expression for three genes (col1a, furin, thbs1, Supplemental Figure S1) in the presence of either wild type, the R398 variants, or the LDS variant. In contrast, there was a significantly reduced expression of these genes in the presence of the TGFBR1 constitutively active variant. Interestingly, expression of furin showed a significant increase over other conditions in the presence of p.R398H, while a trend can be seen for col1a and thbs1. Other TGFB-involved genes that can be measured in 3T3 cells did not differ from baseline conditions (KLF6, SPTBN1, FSTL1, FN1). Given that we see a general upregulation in responding genes for all TGFBR1 conditions, except for the T204D, we suspect this might be a more general mechanism rather than a mutation-specific one, on top of the limitation that these experiments were performed in vitro. To obtain a robust insight into the individual molecular responses of the TGFBR1 variants, one would need to engineer knock-in mice or obtain IPSC from patients.

## 4. R398 TGFBR1 Is Surface Exposed

**Figure 4:**
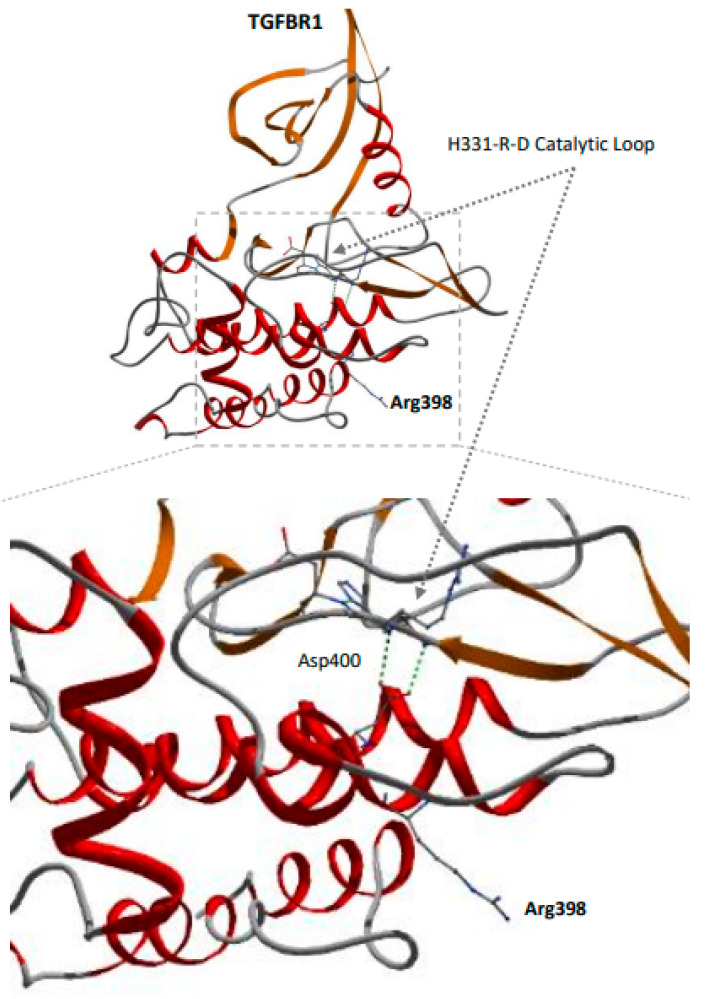
Crystal structure of the TGF-beta receptor 1 (ALK-5) [13]. Asp400 is in hydrogen-bond distance from the backbone amide groups of the key catalytic loop residues His331-Arg332-Asp333 [14], helping in stabilizing this key region of the receptor. The Arg398 is located away on the outside of the protein and away from the catalytic loop.

As the R398 variants are located in close proximity to a known pathogenic location (D400), the patients involved have different phenotypes, and we also looked at the crystal structure of TGFBR1 [13]. The Asp400 is in a critical position, inside the protein, where its side-chain carboxyl group is positioned at hydrogen-bond distance from the backbone amide groups that are part of the key catalytic loop residues His331-Arg332-Asp333 (Figure 4) [14]. In this respect, Asp400 probably plays a significant role in stabilizing this key region of the receptor. A change of this residue to a Gly would result in the complete loss of this salt bridge and thereby possibly affect the catalytic domain’s position and the activation loop. In contrast, the consequences of the Arg398Cys/His changes are somewhat harder to explain based on their positioning. Arg398, based on the 3D structure, has a surface exposed side chain, directed away from the active site. The observed effect of substitution for either a cysteine side chain, generally considered hydrophobic with polar characteristics, or histidine with its charged imidazole side chain, would suggest that the effect is less direct, perhaps inducing a more subtle structural shift that in some way facilitates either enhanced protein-protein interaction or enhanced catalytic activity. In conclusion, there is evidence for mechanistic differences between the pathogenic LDS p.D400G variant, which is located close to the active loop, and the R398 variants, which are located facing away from the active site.

## 5. Discussion

We report here on two families with various forms of congenital heart disease without aortopathy, in which we identified rare variants in *TGFBR1*. While these variants target the same amino acid (R398), the amino acids introduced by each variant are different. We argue that these variants cause disease in both families, as the variants are extremely rare, co-segregate with the disease in both families, occur in an evolutionary heavily conserved region, and display altered TGFBR1-smad signaling activity.

Transforming growth factor-beta (TGFβs) are a family of multifunctional cytokines that affect diverse cellular pathways, including proliferation, migration, synthetic repertoire, and apoptosis, and play crucial roles in the development and maintenance of various organs, including the heart and aorta. It is early in vertebrate embryogenesis that a left–right axis is developed. In the heart, associated vessels, and inner organs, asymmetry is essential for the spatial arrangement and morphology of the organ. Secreted growth factors of the TGFβs family play a pivotal role in the induction of mesoderm and endoderm and the determination of the left–right asymmetries [15,16]. In recent years, there has been emerging evidence that pulmonary hypertension (PH) is associated with variants in the receptor for TGFβs. Studies have shown that pulmonary remodeling of the arteries, the main pathological alteration in PH, is an established risk factor in patients with atrial and/or ventricular septal defects [17,18,19].

To initiate its function, TGFβs ligands need to bind to its receptors, i.e., TGFBR1 and TGFBR2. These receptors are transmembrane serine/threonine kinase receptors. In general, the TGF-β ligands initially bind to TGFBR2, which induces dimerization and enables the TGFBR2 homodimer to form a stable hetero-tetrameric complex with the TGFBR1 homodimer. This leads to the activation of several downstream pathways, which include the canonical SMAD-dependent pathway (SMAD2 and SMAD3) and several non-canonical (SMAD-independent) signaling pathways, such as the p38 MAPK/JNK pathway [20]. In mice, TGFBR1 expression is present specifically in the great arteries and the heart, and enriched in the smooth muscle cell layer of the aorta [21]. TGFBR1 knockout mice are embryonic lethal around E10.5 because of aberrant vasculogenesis in the yolk sac [5]. In humans, heterozygous variants in *TGFBR1* have been associated with a rare genetic disorder known as Loeys–Dietz syndrome (LDS), which shares overlapping, but distinct phenotypic characteristics with Marfan syndrome [22]. Major LDS cardiovascular manifestations include generalized thoracic aortic aneurysm and dissection (TAAD) [12,23,24]. To date, most of the reported familial cases identified with unique variants in TGFBR1 were characterized by extracardiac malformations and genetic syndromes with an autosomal dominant pattern of inheritance. Most pathogenic variants of *TGFBR1* in LDS are missense substitutions of evolutionarily conserved residues within the serine/threonine kinase domains that have been verified in vitro to be associated with loss of function, or are predicted to do so [23,25]. Remarkably though, these loss-of-function TGFBR1 variants actually lead to activated TGF-β signaling in the aorta of these patients, which is referred to as the TGF-β paradox [26]. The exact mechanism behind this is still under investigation.

In contrast, we report here on two *TGFBR1* variants in two families in whom affected members have various forms of congenital heart disease, but no aortopathies, nor signs of extracardiac phenotype or phenotypes compatible with LDS. Strikingly, our two R398 TGFBR1 variants are only two amino acids away from a known loss-of-function LDS variant, but our variants demonstrate gain of function in SMAD signaling in one cell line and loss of function in another, while the LDS variant is loss of function in both. We hypothesize that this contrast in molecular function and patient phenotype might be due to the fact that the amino acid affected in our families is located on the outside of the protein, away from its catalytic site, while the LDS variant is located in the heart of the active site of TGFBR1. Moreover, TGF-b signaling is pleiotropic and depends on contextual determinants such as the type and developmental state of the target cell and the presence of response-modifying signals [27]. It is therefore conceivable that different (pathogenic) TGFBR1 variants can indeed result in different phenotypes. In general, it is clear that our variants and the LDS variant behave significantly differently in comparison to wild-type TGFBR1 in the in vitro assays, and from this, in combination with the co-segregation, evolutionarily conserved position and rarity of the variants, we conclude that the R398 variants are also pathogenic.

Another interesting aspect of this study is the difference in penetrance between the p.R398C variant and the p.R398H variant, in that only p.R398C gives a phenotype in a heterozygous state. We hypothesize that, as the normal arginine at this position is positively charged, its substitution to histidine (as seen in Family B) might have a smaller overall consequence, as histidine also has a charged side chain, while cysteine (as seen in Family A) is polar. This would be in line with the inheritance and penetrance observed in both families, in that p.R398C on its own, in a heterozygous state already results in a phenotype, while the p.R398H only demonstrates a phenotype in the recessive state. There are also some phenotypic differences between the two reported families, in that Family A also has an electrical phenotype. The reason behind this is unclear, given that both variants behave similarly in our assays (with the exception of furin levels). One reason, as stated earlier, might be a difference in protein-protein interactions for the two R398 variant TGFBR1 proteins based on the resultant amino acid; alternatively, the phenotypic difference could also be ascribed to differences in the genetic background, which is known to play a large role in CHD [28]. Indeed, from transgenic mice studies, it is known that CHD severity and the spectrum of defects resulting from a pathogenic mutation is strongly dependent on the genetic background (e.g., 24% of C57BL6/J mice and 70% of FVB mice carrying a Gata4 mutation showed no phenotype in a study by Rajagopal et al. [29]). Taken together, we hypothesize that the overall difference in phenotype in our patients compared to LDS patients is due to the differences in how each variant impacts SMAD signaling differently, as well as the location of the variants in the protein. Further research into the mechanism(s) behind the altered TGFBR1 function and perturbed (cardiac) development is needed.

In conclusion, we describe two families with various forms of inherited non-syndromic CHD without aortopathies. Both families have a rare missense variant in *TGFBR1* on the same amino acid, though these variants led to different substituted amino acids. Interestingly, both variants demonstrated a disturbed TGFBR1-smad signaling activity. These findings further expand the phenotypic scope of (likely) pathogenic TGFBR1 variants and warrant consideration of causative *TGFBR1* variants in CHD patients without aortopathies.

## Data Availability

The WES data in this study are available on reasonable request from the authors.

## Supplementary Materials

The following supporting information can be downloaded at: https://www.mdpi.com/article/10.3390/jcdd10110455/s1, Figure S1. qPCR of various genes involved in TGFB signaling in 3T3 cells that were stimulated for 24 h with TGFB1. * *p* ≤ 0.05 in relation to WT TGFBR1, # *p* ≤ 0.05 in relation to pcdna (background), n = 6 per condition; Figure S2. Western blot for phosposmad2/3 and b-actin in 3T3 cells that were unstimulated, stimulated for 1 h or 24 h with TGFB1. 3T3 cells were either untransfected (pcdna) or transfected with wild type or the R398 TGFBR1 variants; Table S1: General clinical and genetic characteristics of members of Family A; Table S2: Aorta clinical and genetic characteristics of members of Family A; Table S3. Clinical characteristics of the affected members of Family B; Table S4. Various annotations, pathogenicity predictions, and ACMG classification of the two variants of Families A and B.
