# Supplementary material for: TGFBR1 Variants Can Associate with Non-Syndromic Congenital Heart Disease without Aortopathy
Source: J Cardiovasc Dev Dis. 2023 Nov 9;10(11):455. doi: 10.3390/jcdd10110455 (PMC10672196; doi:10.3390/jcdd10110455)
Supplement: Supplementary file 1 [file jcdd-10-00455-s001.zip › jcdd-2683819-supplementary.pdf]

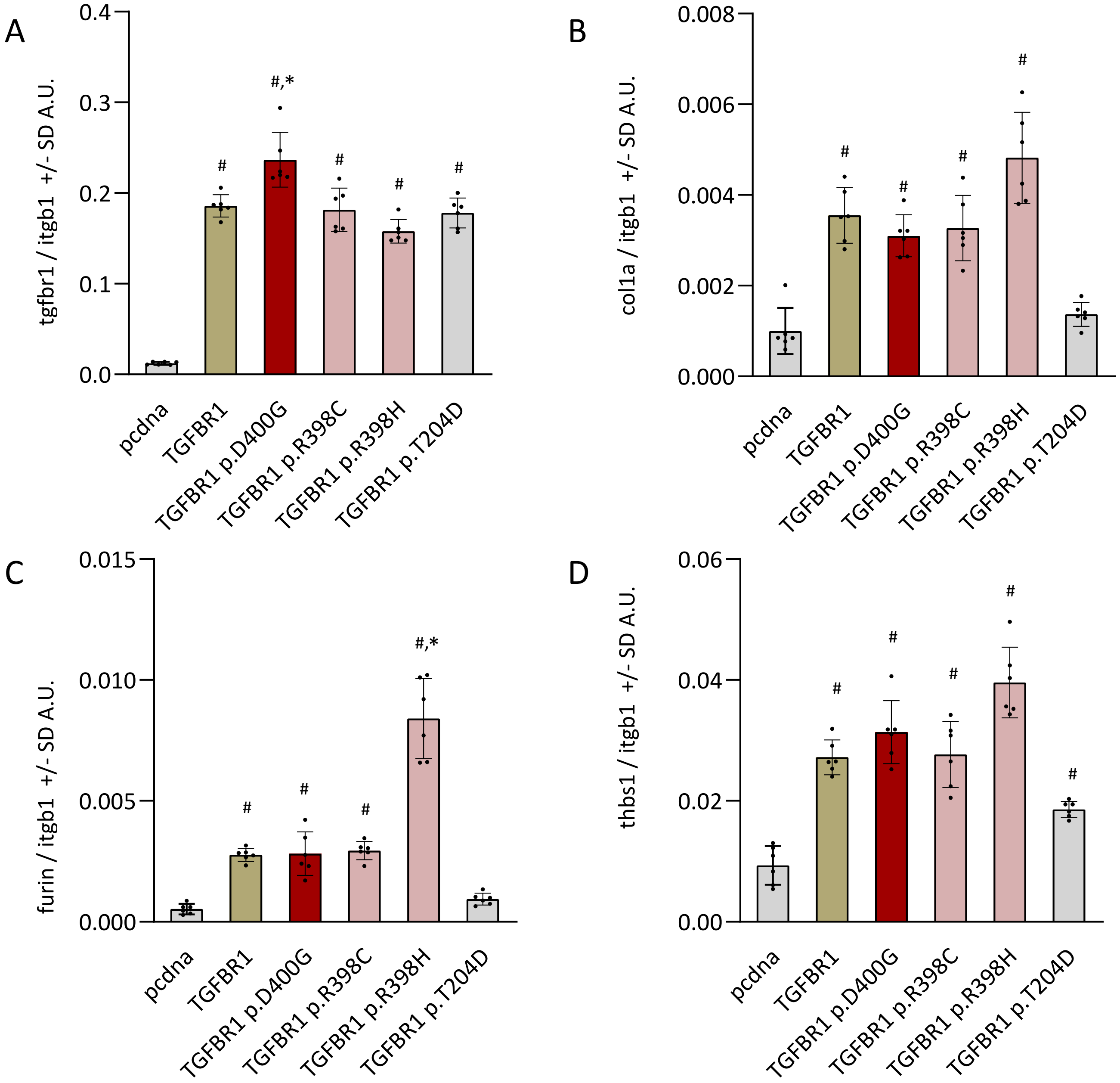

Supplementary Figure S1. qPCR of various genes involved in TGFB signaling in 3T3 cells that were stimulated for 24h with TGFB1. *P ≤ 0.05 in relation to WT TGFBR1, #P ≤ 0.05 in relation to pcdna (background), n=6 per condition.

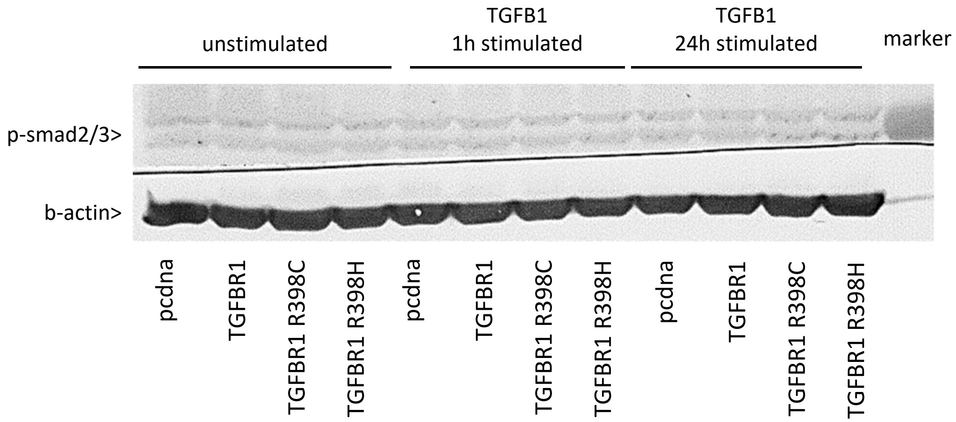

Supplementary Figure S2. Westernblot for phosposmad2/3 and b-actin in 3T3 cells that were unstimulated, stimulated for 1h or 24h with TGFB1. 3T3 cells were either untransfected (pcdna) or transfected with wildtype or the R398 TGFBR1 variants.

Patient characteristics family A	Sex/age	Age at diagnosis of CHD and/or low atrial rhythm	Age (years) at last available ECG	Low atrial rhythm	Rhythm/conduction disturbances	*Congenital heart defects*
II-1	F/83	74 years	77	+	AF	—
II-2	M/89	84 years	84	±	Bradycardia, atrial/ventricular arrhythmia, AFa	—
II-3	M/87	78 years	80	−	Bradycardia, junctional escapes, AF, complete RBBB	—
III-3	F/57	50 years	57	+	Paroxysmal SVT	LSVC, aberrant right subclavian artery
III-4	M/50		43	−	—	—
III-5	M/47	40 years	40	+	First-degree AV block, incomplete RBBB	—
III-6	M/61	10 years	36	±	Complete RBBB	Tetralogy of Fallot, LSVC
III-7	M/59		58	−	Sinus bradycardia	—
III-8	M/54		53	−	—	
III-9	F/47	4 years	38	+	Bradycardia, atrial arrests with AV junctional escapes, intermittent complete AV dissociation	Suspicion of abnormal atrial appendage
III-10	M/60	52 years	52	+	Bradycardia	—
III-11	F/52		45	−	—	—
III-12	F/50		43	−	—	—
IV-1	F/28	21 years	28	+	—	—
IV-2	M/25	24 years	24	±	—	—
IV-3	F/23	2 months	16	+	Incomplete RBBB	ASD II, membrane in left atrium, LSVC, absent brachiocephalic vein
IV-4	F/17	Birth	16	−	Chronic SVT; atrial extrasystoles	—
IV-5	F/28	4 years	28	+	Incomplete RBBB	Systolic murmur
IV-6	M/34		33	−	—	—
IV-7	F/31		24	−	—	—
IV-8	F/18	<0.5 years	18	+	Incomplete RBBB	Incomplete AVSD, ASD II, LSVC draining into left atrium, absent coronary sinus, absent brachiocephalic vein
IV-9	F/36	32 years	32	+	—	—
IV-10	F/33	<0.5 years	33	+	Incomplete RBBB	Incomplete AVSD, common atrium, bilateral left atrial appendages

Table S1

Family A pedigree ID as reported in vd Meerakker 2009	Affected as in vd Meerakker 2009	Carrier of p.R398C	Ultrasound data aorta?	Heart/aorta data extracted from clinical records	Ultrasound details if available
II-1	y	y	y	aorta not mentioned, echo heart performed 79y	Ultrasound at age 79 was considered normal, aortic dimensions not specifically mentioned
husband of II-1	n	n	y	ct thorax: aorta mentioned, but not described as widened 77y	Cardiac ultrasound at age 85 considered normal, no dimensions mentioned
II-2	y	y	n	no imaging performed 86yr	n/a
wife of II-2	n	n	n	no clinical records	n/a
II-3	y	y	y	deceased 2005; echo 80y, aorta 41 mm	Cardiac ultrasound at age 80; ascending aorta 32mm
III-3	y	y	y	2017 dilated sinus coronarius	Cardiac ultrasound age 70, normal, no dimensions mentioned
III-4	n	n	y	2002: echo performed. Aorta not measured	Cardiac ultrasound age 43 normal, no dimensions mentioned
III-5	y	y	y	2002: aorta 31 mm (40 jr)	Cardiac Ultrasound aortic root 31 mm, age 40
wife of III-5	n	n	n	no imaging	n/a
III-6	y	y	n	no clinical records	n/a
III-7	n	n	n	no imaging	n/a
III-8	n	n	n	2009 visited cardiologist, no further info	n/a
III-9	y	y	n	2016 echo performed: no mention of aneurysma	n/a
husband of III-9	n	n	n	married in, no records	n/a
III-10	y	y	n	no clinical records	n/a
wife of III-10	n	n	n	married in, no records	n/a
III-11	n	y	y	2003 aorta 30mm	Cardiac ultrasound age 46, aortic root 30mm
III-12	n	n	y	2003 aorta 28 mm	Cardiac ultrasound age 44, aortic root 28mm
III-13	n	n	y	2003 aorta 28 mm	Cardiac ultrasound age 46, aortic root 28mm
IV-1	y	y	y	2003: 29 mm (21jr)	ultrasound age 21, aortic root 29 mm
IV-2	y	y	n	2009: normal heart, no dimensions mentioned	n/a
IV-3	y	y	n	ASD type II, no specific information on aorta	n/a
IV-4	y	y	n	no imaging	n/a
IV-5	y	y	y	2002; aorta 27 mm	Cardiac ultrasound age 20, aortic root 27mm
IV-7	n	n	y	2002: aorta 30,mm	Cardiac ultrasound age 24, aortic root 30mm
IV-8	y	y	y	2014: coractatio aorta Aorta ascendens: 27 mm.	Surgery for incomplete AVSD, mild aortic coarctation, MRI ascending aorta 27mm, coarctation in the descending aorta, 13x15mm, age 23
IV-9	y	y	n	2006: echo performed, aorta not measured	n/a
IV-10	y	y	n	Multiple echos, aorta diameter not mentioned	n/a

Table S2

Patient characteristics family B	1st Child (proband)	2nd Child
Gender	M	F
Age	7y	21months
Weight	17.6kg	8.5kg
Hight	119cm	74cm
Clinical features	Large ASD, large inlet VSD, dysplastic and straddling mitral valve, Tricuspid valve hypoplasia and right ventricular hypoplasia	Complete AVSD with small left atrioventricular valve and common atrium
Aortopathy	Competent aortic valve, no dissection	Competent aortic valve, no dissection
Cardiovascular Symptoms	Post-surgery accelerated junctional rhythm	Depressed cardiac function and moderate common atrioventricular valve regurgitation
Surgical Procedures	Pulmonary artery banding at 3 months of age then cavo-pulmonary shunt (BCPS) and tightening of the pulmonary artery band at the age of 19 months then completion of Fontan operation was done at the age of 2.5 years.	Pulmonary artery banding
Dysmorphism	None	None
Motor development	Normal	Normal
Mental Development	Normal	Normal
Neurological Abnormalities	Normal	Normal

Table S3

ID	Chromosome	Position (hg19)	Ref	Alt	Transcript	Aminoacid change	cDNA change	CADD PHRED	CADD RAW	SIFT	gnomAD AF	rsID	PFAM domain	ACMG/AMP classification	ACMG/AMP evidence
variant family A	9	101908828	C	T	ENST00000374994.4	p.R398C	c.1192C>T	23.8	3.093413	tolerated	0/251180	NA	Protein kinase domain	Pathogenic (II)	PS3, PS4, PP1-S, PM2, PP1-M, PM1, PP1
variant family B	9	101908829	G	A	ENST00000374994.4	p.R398H	c.1193G>A	24.2	3.256756	deleterious	1/251180	rs200657153	Protein kinase domain	Pathogenic (II)	PS3, PS4, PP1-S, PM2, PP1-M, PM1, PP1

Table S4
